# Toward security-aware portable sequencing

**DOI:** 10.1038/s41467-025-66024-z

**Published:** 2025-11-10

**Authors:** Carson Stillman, Jonathan E. Bravo, Christina Boucher, Sara Rampazzi

**Affiliations:** Department of Computer and Information Science and Engineering, Herbert Wertheim College of Engineering, University of Florida, Gainesville, FL USA

**Keywords:** Policy, Computational platforms and environments, Data acquisition

## Abstract

Portable genome sequencers are revolutionizing genomic research. However, their reliance on external systems introduces new vulnerabilities that threaten the security of these sequencers. By employing zero-trust principles throughout the sequencing workflow, we can enhance the security of portable sequencing technology.

## Redefining security for portable sequencers

**Fig. 1:**
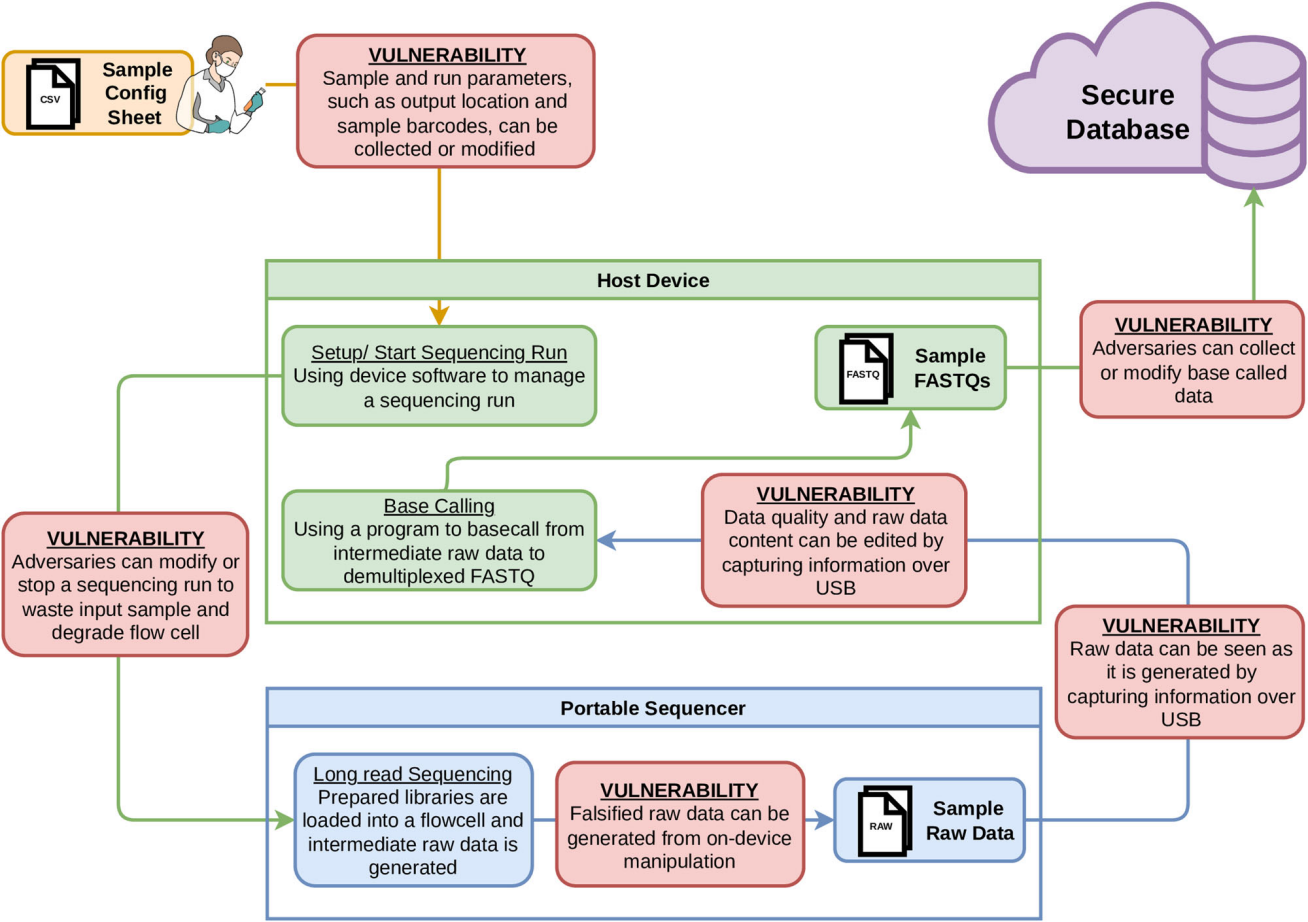
Portable genome sequencing device connected via USB to an external host. The red squares highlight security risks to confidentiality, integrity, and availability when the host machine is compromised.

The emergence of portable genome sequencing technology has the potential to transform the way biological samples are collected and analyzed. We define *portable sequencing* as a compact instrument that acquires raw signals on-device but relies on an external host (e.g., a laptop or desktop) for basecalling and further elaborations, whereas standalone sequencing refers to self-contained benchtop platforms that integrate signal conversion and computation within the device. These “sequence anywhere” devices are “palm-sized platforms designed for use in the lab or field” (https://nanoporetech.com, Accessed September 22, 2025). For example, in a seminal field deployment between 2014 and 2016 West African Ebola epidemic, a portable nanopore system was transported to Guinea and used to perform real-time genomic surveillance, producing results within 24 h of sample receipt, and sequencing runs as short as 15 min on 142 clinical samples. These findings demonstrated that genomic surveillance can be established rapidly in resource-limited settings and can inform outbreak tracking and response logistics without relying on centralized laboratories^1^. However, these new capabilities have the potential to open new attack vectors and vulnerabilities for adversaries who wish to undermine the security and privacy of the sequenced data. Unlike traditional standalone laboratory equipment, these devices often lack the computational capacity to convert raw signal traces into sequence data. Instead, they use a host machine to perform the necessary signal processing and basecalling tasks. The decoupling of sequencing hardware from computing systems has the potential to open up a range of security vulnerabilities by broadening the attack surface to threats that were not considered before when used outside secure laboratory environments, as illustrated in Fig. 1. In other words, there are more avenues for attackers to attempt to gain access and alter data, such as compromising host machines to manipulate the elaboration, and exploiting insecure communication channels between sequencers and hosts for eavesdropping.

These potential threats highlight the need for coordinated work between the security and bioinformatics communities, expanding genomic data protection from purely post-sequencing measures to end-to-end, secure-by-design practices. In recent decades, research has been devoted to mitigating the privacy risks of DNA data storage in online databases and sharing after the sequencing process is complete. However, ensuring the security of the initial stages of the sequencing workflow, where genetic material is processed and data is generated, has become increasingly critical. Both research studies and the recent Illumina recall^2^ have shown how unauthorized users can exploit design vulnerabilities to take control of the sequencing process remotely, altering configurations to affect the results of genomic data, including evading detection or spreading malware^3^. The resulting risks for users may include the targeting of individuals based on genetic traits, financial fraud, identity theft, and the unethical use of genomic information without consent, while severe consequences can involve altered bioinformatic diagnoses, ultimately compromising patient health and safety.

From a cybersecurity perspective, these concerns are further amplified by the increasing mobility and decentralization of these portable sequencing technologies. As these devices can be connected to potentially insecure networks through their host devices when used outside the conventional laboratory environment, the security and trustworthiness of sequencing processes can no longer be based solely on laboratory security practices. Instead, security must be established and guaranteed from the design of the sequencer, extending to all components of the sequencing workflow. This *ground-up* approach presents nontrivial challenges, as traditional security measures typical of laboratory equipment may not apply to resource-constrained portable sequencers when used in the field, and secure operations can still pose significant privacy risks when handling genomic data.

This paper outlines emerging vulnerabilities specific to portable sequencing workflows, demonstrating three design flaws found on a portable Oxford Nanopore sequencing device. Oxford Nanopore Technologies has since implemented mitigation strategies for the discovered vulnerabilities, which we describe in detail in the “Observed vulnerabilities” section. By documenting these now-mitigated issues, we promote transparency, facilitate third-party verification, and distill generalizable guidance that improves secure-by-design practices. Finally, we propose a zero-trust security approach designed to address confidentiality, integrity, and availability from the moment data is generated to its secure storage. Coordinated efforts with platform providers, such as Oxford Nanopore’s ongoing work to identify and mitigate vulnerabilities, are essential to translate these recommendations into deployable, secure-by-design portable sequencing systems. In summary, as portable sequencing devices gain widespread adoption, there is an urgent need to reevaluate the assumptions that underlie current genomic data protection strategies.

## Vulnerabilities introduced by portability

NIST outlines security practices for securing genomic data across acquisition and storage in centralized laboratories^4^. In combination with prior research on secure storage and inter-organizational sharing^5^, this body of work defines the safeguards for the storage and sharing of genomic data. Such techniques include the use of federated learning and other privacy-enhancing technologies (PETs), such as differential privacy, that allow organizations to extract information without revealing specific details about individuals^6^. These security practices focus on the assumption that sequencing data is generated and stored on standalone sequencing devices, with the sequence data transferred through a secure network or to external hard drives and workstations when sequencing operations are concluded. In contrast, current portable sequencers are engineered to offload their computational tasks to auxiliary host machines. This necessitates the storage and exchange of unprocessed signal data between the sequencer and the host, during and after computational processes. Consequently, this transfer could potentially expose raw or un-base-called data. Furthermore, unlike standalone sequencers, portable sequencing devices can perform sequencing operations outside laboratory networks and, when doing so, rely on the host to establish an Internet connection for data sharing. This connection must be active by default on current sequencers to facilitate maintenance and telemetry between the sequencer’s software and cloud services. Nonetheless, this connection also exposes the system to potential cyberattacks.

A common but flawed assumption is that security can be ensured simply by disconnecting the device from the Internet during the sequencing procedure. Unfortunately, this approach does not recognize that a host machine, which can be connected to the Internet at any time, remains vulnerable to security breaches and malware. Thus, merely halting the Internet connection before or during sequencing does not eliminate the risk of data exfiltration and manipulation due to a compromise in the host machine.

Collectively, these observations underscore that the portability of sequencing devices, while enhancing accessibility and operational flexibility, fundamentally redefines the security landscape, necessitating a reevaluation of trust assumptions, host-machine dependencies, and protection mechanisms traditionally accepted in controlled laboratory environments. A clear example is when company policies like Bring Your Own Device allow users to connect their personal computers, not managed by organizations’ IT security groups, to the sequencers to perform job duties locally or remotely. An employee in a laboratory might unknowingly fall victim to phishing attacks by clicking on a malicious link in a seemingly legitimate email, thus compromising their device. This compromised personal computer, once connected to the sequencer, can be used to manipulate sequencing data during elaborations or allow unauthorized access to sensitive sequencing data while the user is unaware of the compromise. To the best of our knowledge, these vulnerabilities have not been exposed in the wild, meaning they have not been observed being actively exploited in current real-world systems or environments. Further research and empirical validation are necessary to assess the extent of the threat. A summary of these potential exploitations and their impact is summarized in Table 1.

**Table 1:** Overview of vulnerabilities in portable sequencing devices with examples and potential impacts

Vulnerability	Examples of exploitation	Potential impact	Solutions
(I) Flaws in remote authentication when connected outside laboratory networks	• MitM attacks and online password guessing • Exploitation of weak or lack of authentication processes to connect to the sequencer	• Unauthorized access to sequencing data stored in the host machine and remote control of sequencing processes.	• Implement IAM roles for access control (local and remote) • Use passwords, multi-factor authentication, or passkey to connect to the sequencer in addition to the host
(II) Flaws in the sequencing software on the host machine	• Injection of harmful commands via the user interface • Bypassing login screens due to weak security measures when the host is compromised (malware)	• Unauthorized access or control of the sequencer • Viewing, theft, modification, or deletion of sequencing data and configurations • Creation of false base-called data	• Employ public-key cryptographic techniques to prevent data falsification and unauthorized access on the host machine • Verify all data requests for malicious patterns
(III) Flaws in the host machine	• Installation of malicious software (e.g., via phishing or USB keys) • Ransomware attacks encrypting files of the sequencer and host	• Locked or inaccessible sequencing data • Financial or operational losses due to ransom demands • Unauthorized control of the sequencer, manipulation of operations, and data eavesdropping	• Establish applications’ privileges and user permissions to access and modify sequencing data (least privilege). • Encrypt data during storage in the host
(IV) Flaws in host-sequencer communication and data sharing	• Interception or disruption of messages between host and sequencer (malware) • Exploiting weak USB protocols to modify raw data	• Unauthorized view, alteration, deletion, or injection of false raw data • Degradation of sequencing output quality • Interruptions in sequencing processes causing sample and flowcell loss	• Perform integrity checks and checksums to identify alteration or corruption of data during each information exchange. • Encrypt data during transmission to the host device
(V) Flaws in the software on the sequencer	• Denial-of-service attacks by overloading the sequencer memory through the host • Exploitation of inadequate storage protection to alter data or configurations in the sequencer	• Inoperability of the sequencer for authorized users • Raw data alteration, deletion, or creation of false data for sequencing output manipulation	• Apply sanitization to remove harmful input entered by unauthorized users. • Monitor system functioning for malicious patterns

### Threats to confidentiality

Confidentiality is the property that information is disclosed only to authorized entities; it requires controls that prevent unauthorized access or exposure during storage, processing, and transmission. For example, without proper protection mechanisms to ensure secure data transmission (i.e., encryption), the sequence data that are unconditionally transmitted to the host machine for elaboration can be potentially intercepted by unauthorized users. This can include either eavesdropping on intermediate data or exfiltration of the final sequencing data post-basecalling. Confidentiality can also be violated when unauthorized users gain access to the sequencer or the data stored in the host machine when compromised (e.g., the host is infected by malware). Portable sequencing devices often allow users to connect to the sequencer over the network, only using password-based authentication in the application installed on the host machine. This enables users to directly control sequencing runs as well as to monitor in-progress experiments remotely. If host devices are connected to insecure or public networks in the field, the risk of data breaches increases. For example, malicious entities can adopt common phishing techniques or Man-in-the-Middle (MitM) attacks that can bypass private network protection, such as VPNs, to steal passwords and access the sequencer, especially when weak authentication protocols are used. This has been observed in practice with the recent recall of Illumina sequencers, where the associated software exposed connection ports that allowed unauthenticated and unauthorized remote users to connect to the sequencer, alter settings and configurations, and access sensitive data^2^.

### Threats to integrity

In addition to confidentiality, integrity ensures that data are protected from unauthorized manipulation. Integrity is the security property that preserves the accuracy, completeness, and original state of data throughout every phase of its lifecycle, including generation, collection, storage, processing, analysis, archiving, and destruction. In the case of genomic data, violations of this property can result in an adversary being able to manipulate the base-called sequence, evading traditional consistency controls, which can result in misleading or invalid genomic interpretations with potential clinical consequences when used for clinical purposes. These threats might arise in portable sequencers from erroneous or misconfigured privilege authorization in their programs and applications, when used outside regulatory and manufacturer guidance, or outside controlled laboratories. In this context, *privilege* refers to the permissions granted to a system or program to perform specific operations or access certain resources. The host machine might be granted additional privileges to access and modify raw sequencing data for different elaboration purposes (i.e., basecalling operations). If the host is compromised, adversaries could take advantage of this permission to execute unauthorized operations and manipulate genomic sequences^3^.

### Threats to availability

Finally, adversaries can disrupt the sequencing workflow by taking advantage of design flaws in the host machine software and weak authentication techniques for the host-sequencer connection. Availability is the security property that ensures that services can be used and reached when needed without being intentionally blocked. For example, sequencing runs can be halted, paused, or disrupted mid-sequence, or be flooded with excessive requests, overwhelming the sequencer’s limited processing capabilities and rendering it unusable. This is what is known as a Denial-of-Service (DoS) attack, which can be perpetrated by unauthorized users. Furthermore, unprotected portable sequencers can be the victim of ransomware attacks, which can encrypt data read on the sequencer through the host machine until a ransom is paid. In 2024, 92% of healthcare organizations were the target of a cyberattack, and 69% reported at least one cyberattack that disrupted patient care (see 2024 Ponemon Healthcare Cybersecurity Report: https://www.proofpoint.com/us/resources/threat-reports/ponemon-healthcare-cybersecurity-report Accessed: 2025-04-28). Such disruptions can result in postponed diagnostic decision-making, compromised clinical workflows, and loss of irreplaceable biological samples, each of which may have significant consequences for patient outcomes.

### Observed vulnerabilities

We have performed a preliminary vulnerability analysis on the popular Oxford Nanopore MinION Mk1B portable sequencer and the associated MinKNOW software to understand the extent of the threat. Our results uncover three vulnerabilities that can lead unauthorized users to remotely access the sequencer and pursue Denial-of-Service and Ransomware attacks. We followed standard security disclosure practice by reporting the vulnerabilities through the Vulnerability Information and Coordination Environment (VINCE), a centralized coordination platform developed by CERT^7^, and sponsored by the US Cybersecurity and Infrastructure Security Agency as part of the Coordinated Vulnerability Disclosure Program (see Coordinated Vulnerability Disclosure Program: https://www.cisa.gov/resources-tools/programs/coordinated-vulnerability-disclosure-program Accessed: 2025-09-28). An independent coordinator facilitates communication between the vendor (here, Oxford Nanopore Technologies) and the reporters to ensure dissemination of vulnerability information and associated mitigations. Under the commonly used 90-day coordinated disclosure window, vendors have time to investigate and mitigate the reported vulnerabilities before the details are publicly released. After this period, CISA, in coordination with the vendor and reporter, discloses the vulnerability to the public by issuing a CVE record in the CVE public database and a public advisory. A CVE, which stands for Common Vulnerabilities and Exposures, typically contains a description and data about a specific cybersecurity vulnerability, including a unique CVE identifier, references to technical details, and the remediation steps provided by the vendor. Hence, the vulnerabilities we have identified were disclosed to the vendor under the responsible disclosure process and assigned three CVEs (CVE-2024-35585, CVE-2025-54808, CVE-2025-10937).

#### Lack of secure authentication for remote access

The first vulnerability that we observed is a flaw in the authentication mechanisms to access the sequencer through a remote connection. To observe, start, and modify sequencing runs remotely, MinKNOW software, which handles sequencing control for all Oxford Nanopore sequencers, enables users to connect to their sequencer via a remote host machine (e.g., a laptop). We observe that the device requires users to be logged into a valid Oxford Nanopore account on the host machine software and use the IP address of the sequencer to access the device. However, IP addresses are publicly visible identifiers used for network communication, which cannot be encrypted; thus, they can be easily intercepted or spoofed by malicious users on the same network. For these reasons, they are considered unsuitable for use as authentication credentials (CWE-291: Reliance on IP Address for Authentication: https://cwe.mitre.org/data/definitions/291.html Accessed: 2025-04-22). The analysis is performed from the perspective of an unauthorized user who is successfully able to register an Oxford Nanopore account and knows potential sequencing devices connected to the target network. Based on this, we demonstrate that it is possible to create and verify an account using a temporary email address and remotely connect to a victim sequencer after a quick IP address scan using open-source software for network mapping. This basic exploitation method is sufficient to obtain access to the sequencer, as authenticating on the software in the host automatically grants access to the device, without further verification mechanisms. Unauthorized users can then observe as the data are sequenced, start/stop sequencing runs, and modify the data output location on the host machine. The interruption of in-process sequencing runs can result in wasting samples and flow cells, while modifying the output location can redirect the sequencing data to an unauthorized network-attached drive that malicious users can use to exfiltrate sensitive data before uploading it to secure online databases.

In response to this vulnerability, Oxford Nanopore has released a software update issued through MinKNOW Software Version 24.06 on the 31st of July 2024 with user notification delivered via in-software changelog and release notes published on the Oxford Nanopore Community. This update includes remote access being disabled by default. Users must explicitly enable it to operate remotely.

#### Insecure local-access token

The second vulnerability is also associated with the MinKNOW software application and can compromise the confidentiality and integrity of the sequenced data, as well as the availability of the sequencer in the case of a compromised host device or the presence of a malicious insider. Through standard software analysis, we observed that the MinKNOW software stores an authentication token in a temporary folder on the host machine. The token allows access to the sequencer via a local network connection to perform privileged functions such as adjusting the sequencer settings or retrieving sequencing data. This token-based authentication scheme is typical for client-server-based software architectures and is secure as long as the token is kept private^8^. Storing the local token in a temporary folder allows for easy cleanup and management, as the directory is typically cleared on reboot, but makes the token visible to any user and applications on the host machine. Thus, in the case that this machine is infected by a virus or malware, unauthorized users can read the local token and authenticate to the MinKNOW software remotely. Moreover, we discovered that the local token can be used to register a developer token with the MinKNOW software remotely. Designers typically use developer tokens to access the software functionalities, including unlocking specific operations for developers. These tokens can be set with an arbitrary expiration date, allowing remote permanent access to the sequencer without authentication. Oxford Nanopore has released a software update mitigating this vulnerability, which is described in the “Denial of service” section.

#### Denial of service

Finally, we identified a compromise of availability in the sequencer enabled by the same token mechanism described above. When the MinKNOW application starts on a system, it stores the local token in a temporary file before copying it to the final local token file. Malicious software (e.g., malware) installed in the host machine, or a malicious insider, can take advantage of this temporary file to place a lock that prevents the MinKNOW software from writing to the file. When this happens, no valid local token file is generated. Consequently, the software cannot execute any command on the sequencer, preventing sequencing operations from being executed.

In regards to insecure local-access tokens and Denial-of-Service vulnerabilities, Oxford Nanopore has released a software update issued through MinKNOW Software Version 25.05 on the 16th of July 2025, with user notifications via changelog and community release notes. This update disables developer tokens entirely and further reinforces protections for local tokens and DoS attacks.

## Potential solutions

A prevalent misconception is the expectation that the end users of sequencers, such as lab technicians, clinical personnel, and biologists, are also responsible for the overall security of their workflows when outside of their laboratory, during analysis in the field, or remotely. Although in general, security awareness is necessary, this assumption overlooks the complexity of modern connected computer systems and the specialized knowledge required to manage security effectively. Decades of cybersecurity best practices (see Microsoft Security Development Lifecycle: https://www.microsoft.com/en-us/securityengineering/sdl/practices Accessed: 2025-09-28), and seminal research works, such as *Why Johnny Can’t Encrypt*^9^ and *Users are not the enemy*^10^ have long demonstrated the risks of relying on users to configure and maintain security, underscoring the need for transparent, user-focused mechanisms that are enforced automatically, without requiring users to manage complex technical processes. This paradigm acknowledges that insecure user behavior often stems from a lack of technical background in security and poor communication about cybersecurity risks. For example, users might choose to engage in insecure behavior not because they don’t care about security, but because of convenience, such as when security protocols slow down their workflow (e.g., using dual-factor authentication vs. “Remember Me" functionalities to not retype login info). At the same time, a scenario giving the impression that its security mechanisms are perfectly secure at all times is likely to induce careless behavior, since the level of perceived threat is low. Organizations must actively inform users about existing and potential threats to their systems and the sensitivity of the information they contain. Maintaining users’ security awareness over time and through technology shifts and changing scenarios requires balance. Users need clear guidance to avoid leading them to rely on incomplete personal judgment. Security levels should also change as complexity and scenarios evolve, and be transparently linked to the processes and information they protect. This approach has been proven to reduce the risk of human error and make security more effective in preventing data breaches.

In addition to this, another misconception is considering the security of sequencer devices and the general-purpose host machines as a unicum. Specific requirements and security measures must be tailored for the host machine, different from the sequencers, now that they have been fully integrated into the sequencing workflow and can potentially be deployed in the field outside the protected laboratory environment. Vulnerabilities derived from the sequencer’s portability are problematic precisely because the increased scope of attack makes exploitation more likely. Solutions to these new security problems are multifaceted and must evolve as the complexity of the interactions between host and sequencer increases. Here, we argue that protecting the sequencing workflow can be accomplished by adhering to *zero-trust* principles, as described in the Microsoft Security Development Lifecycle, a set of security best practices widely adopted across the modern software industry to integrate security into development processes. Zero-trust assumes no implicit trust in systems or users based solely on their physical or network connection or based on system configurations, even if they were previously verified^11^. In addition, all requests and access to the data should be authenticated and validated before being granted, preventing unauthorized users from inputting malicious commands or code in both the sequencers and the host machine. This is typically realized following a number of core tenets, which include continuous monitoring and evaluation of a device’s status based on a series of parameters and rules, and by ensuring the principle of least privilege, where each device and software component of a process should have only the minimum access and privileges required for them to perform their task, as summarized in Table 1. For example, one of the core tenets of zero-trust revolves around Identity and Access Management (IAM). In a zero-trust IAM setup, each device and user must be properly authenticated, access is strictly controlled according to roles, and all data is encrypted during transmission and storage in the host.

The first step in implementing zero-trust solutions consists of adopting strong authentication mechanisms to control access to the sequencer and the sequencing data on the host machine, both locally and remotely. In addition to passwords, multi-factor authentication or passkey can significantly increase the difficulty for attackers to impersonate legitimate users while preserving confidentiality and availability. For example, common two-factor authentication mechanisms that require users to approve login attempts have been shown to virtually eliminate an entire class of password-stealing/cracking attacks^12^.

Second, data should be secure even if the host is compromised, as it is transmitted from the sequencer to secure online servers or databases using lightweight encryption suitable for low-resource embedded systems. Achieving this goal may also require rethinking the role of the host machine in the sequencing workflow. For example, one can construct a private sequencing pipeline using public-key cryptography. The sequencer can encrypt the raw data before it transmits to the host device, preventing untrusted intermediaries from gaining access to the underlying genetic information. The host machine, considered untrusted, does not have access to the cryptographic keys necessary to decrypt the collected data, preserving confidentiality. Its sole responsibility is to transmit the data over the network, adhering to the zero-trust principle of least privilege, where each component in the workflow has only the access it needs to perform its function.

When data must be used on the host machine, integrity can be ensured by using techniques such as Message Authentication Codes (MACs), which can detect tampering during data sharing and transmission. These codes, often used on resource-constrained devices, work by having both the sender and receiver compute a mathematical transformation (e.g., a hash) on the data. MACs have two key properties: they are non-reversible, meaning an adversary cannot determine the original sequence from the code, and they rarely collide, meaning different data sequences do not produce the same code. These properties allow the receiver to verify whether the received data has been altered by calculating the code itself. The approach does not assume trusted input from workflow components, allowing each system to independently verify data integrity. For example, these techniques have been explored in wireless body area networks, which collect and transmit patient physiological data.

However, implementing a zero-trust architecture in portable sequencing workflows presents different challenges due to the need to balance resource availability, performance, and usability. Techniques such as two-factor authentication are likely to add a burden to legitimate users by requiring interaction with sophisticated authentication mechanisms for both host machine software and the portable sequencer^12^. Moreover, adding techniques such as encryption can incur computation overhead, which might not be sustainable for portable low-resource sequencers. Careful software redesign using data compression algorithms before encryption can alleviate this overhead, ensuring efficient and secure data processing. Another alternative is to use hardware solutions. For example, fast hardware components have been developed to efficiently handle encryption tasks, making it possible to use strong encryption even in low-power, safety-critical medical devices, such as pacemakers^13^.

An extreme solution considered to protect the sequencers from remote unauthorized access is air-gapping, which involves keeping host and sequencing devices disconnected from the Internet at any time, allowing only physical data exchange (e.g., using USB keys). Although this solution is adopted for certain safety-critical medical devices such as pacemakers and neurostimulator programmers, it might not be applicable to portable sequencers designed for field use, which require an active internet connection and the possibility of remote access by legitimate users. Furthermore, researchers have demonstrated the possibility of eavesdropping on air-gapped computers by exploiting electromagnetic, acoustic, and thermal emissions. Another more viable solution adopted by some healthcare providers is associating the device with a dedicated smartphone or tablet, customized to meet specific needs and security requirements, which differs from general-purpose commercial ones for the general public. This approach is typically used for remote patient monitoring and telemedicine.

In addition to zero-trust, blockchain-based solutions have been proposed to ensure robust, immutable audit logging in genomic sequencing workflows. These solutions improve traceability and transparency by securely recording data access logs and usage^14^. Although blockchain technology offers promising avenues for enhancing access control of genomic data, its relevance to addressing the specific security considerations discussed here remains less clear. Challenges such as scalability, computational overhead, regulatory compliance, and integration complexities persist; however, continued research efforts aim to overcome these obstacles.

Finally, security best practices such as regular security assessments in laboratories, periodic software updates, agreements on security expectations and vulnerability management with vendors, and user education on security best practices can help maintain continuous protection as new threats emerge. That also means laboratory members should be on the lookout for anomalies or a lack of proper training on cybersecurity topics in their work routines.

## Conclusion

Although NIST has recently outlined the urgent challenges in the evolving genomic data cybersecurity landscape^15^, their guidance for protecting genomic data does not cover a comprehensive approach for the security of portable sequencing workflows. Sequencing device security is not only an IT issue, but ultimately, it concerns patient safety. The good news is that there is no need to reinvent the wheel, as medical devices and the Internet of Things face similar challenges. Many organizations, such as AAMI, FDA, and others, have developed guidance and roadmaps that can serve as a starting point for a deeper conversation about sequencing device security (Cybersecurity in Medical Devices: Quality System Considerations and Content of Premarket Submissions: https://www.fda.gov/media/119933/download Accessed: 2025-04-29). Our considerations suggest a clear path forward: revisiting security and privacy risk models of sequencing devices to incorporate portability-related threats, and developing a multilayered security approach based on zero-trust principles to address emerging vulnerabilities in portable sequencing technology as their use in the field becomes widespread. To realize this vision of moving toward a fully secure-by-design system for portable sequencing, collaboration is needed between experts in bioinformatics, security, usability, and biomedical sciences.
